# Supplementary material for: Genomic exploration of the journey of Plasmodium vivax in Latin America
Source: PLoS Pathog. 2025 Jan 13;21(1):e1012811. doi: 10.1371/journal.ppat.1012811 (PMC11761655; doi:10.1371/journal.ppat.1012811)
Supplement: S3 Table — To see the correspondence between the parameters and the scenarios, refer to Fig 5a. All distributions are uniform. (PDF) [file ppat.1012811.s013.pdf]

Parameter name	Minimum	Maximum
Population sizes		
Nbot	1	10
Nanc	100	150,000
NCol	10	100,000
NMauri	10	10,000
NEbro	10	50,000
NAncEbro	10	50,000
NbotEbro	1	10
Nanc1	200	150,000
Nanc2	200	150,000
Nghost	200	100,000
Nghostam	10	100,000
NghostEbro	10	50,000
Nghost2	10	100,000
Admixture proportions		
ra	0.01	0.99
ra2	0.01	0.99

Parameter name	Minimum	Maximum
Event times (in generations)		
tdivadm	500	5,500
tbot	1	10
tdivebraf	500	5,500
tdivebram	500	5,500
tdivamaf	750	8,500
tbotebro	1	10
tadmebro	500	6,500
tdiv1/tdiv1b	500	8,500
tdiv2/tdiv2b	750	8,500
tct2	750	7,500
tdivamaf2	750	6,500
tct3	750	7,500
Conditions		
tdivadm<tdivebraf<br>tdivebram<tdivamaf<br>tdivebram<tdivebraf<br>tadmebro<tdiv1<br>tadmebro<tdiv2<br>tadmebro<tdiv1b<br>tadmebro<tdiv2b<br>tdiv1b>tdiv2b<br>tdiv1<tdiv2<br>tct2<tdivamaf2<br>tct2<tdivebram<br>tdivamaf2<tdivebraf<br>tct3<tct2		
