# Supplementary material for: Genomic exploration of the journey of Plasmodium vivax in Latin America
Source: PLoS Pathog. 2025 Jan 13;21(1):e1012811. doi: 10.1371/journal.ppat.1012811 (PMC11761655; doi:10.1371/journal.ppat.1012811)
Supplement: S4 Table — (PDF) [file ppat.1012811.s014.pdf]

Scenarios	Mean percentage of RF classification votes	Standard deviation of RF classification votes
**1**	3.88	0.30
**2**	10.43	1.26
**3**	1.31	0.20
**4**	8.16	0.86
**5**	1.11	0.41
**6**	21.90	1.06
**7**	9.31	0.88
**8**	15.03	1.02
**9**	9.29	0.55
**10**	5.01	0.74
**11**	12.21	0.94
**12**	2.31	0.50
