# Supplementary material for: Genomic exploration of the journey of Plasmodium vivax in Latin America
Source: PLoS Pathog. 2025 Jan 13;21(1):e1012811. doi: 10.1371/journal.ppat.1012811 (PMC11761655; doi:10.1371/journal.ppat.1012811)
Supplement: Attachment — Submitted filename: Answer_Reviews.pdf [file ppat.1012811.s015.pdf]

MiVEGEC Research Unit
(Univ. Montpellier, CNRS, IRD)
911 Av. Agropolis
34090 Montpellier, France

Dear Editor,

Please find enclosed a revised version of our manuscript (PPATHOGENS-D-24-01090) entitled “Genomic exploration of the journey of *Plasmodium vivax* in Latin America” by Lefebvre and colleagues.

We thank the reviewers for their constructive and helpful comments on the manuscript, which significantly helped us improve its quality. We have modified our manuscript accordingly, following the reviewers’ comments as much as possible. The details and point-by-point answers to the reviewers are provided in the attached file.

We hope you will find this manuscript greatly improved and suitable for publication in PLOS Pathogens.

Yours Sincerely,

Margaux J.M. Lefebvre
(on behalf of all authors)

We thank all the reviewers for the quality of their reviews as well as the high level of their expertise, which significantly helped us improve the quality of our manuscript.

Hereafter are our point-by-point responses to the reviewers' comments. We use specific typography to highlight the reviewer's comments (black), our response (blue), and our corrections in the manuscript (tracked change in colors).

Part I - Summary

Reviewer #1: This manuscript by Lefebvre and colleagues describes the analysis of Plasmodium vivax malaria parasite genomic data to understand the manner in which it was introduced to the Americas. The authors analyzed data from 513 previously sequenced P. vivax samples, and contributed high quality sequencing data from 107 newly sequenced samples, many of which come from previously undersampled (or unsampled) populations, such as Mauritania, Cameroon, Comoros, Sudan, and Central African Republic. With these data, the authors suggest that the current reigning hypothesis (that American P. vivax populations represent an extirpated European clade brought across the Atlantic in the colonial period) is too simple. They propose that the American P. vivax populations exhibit ancestry from W. Africa, as well potentially from other regions such as South Asia.

The authors make the most of sometimes thin data (eg fewer than 10 samples from many countries). The dataset they have assembled reflects similar global population structure as observed in the Pv4 (2002) publication, as well as predecessor publications of comparable global Pv genome datasets. The authors analyse the data more deeply with regard to population structure and recent demography than the Pv4 manuscript, however, making these results of interest to a wide audience. The manuscript is very clearly written.

R1. We thank the reviewer for their summary. While this is true that some sampled populations/locations are "thin", analyzing whole genome data provides access to thousands, and even millions of genetic markers (SNPs). Physically unlinked SNPs in the genome provide as many independent "samples" to probe the evolutionary history of a population or a species, back to its most common ancestor (Hahn, 2018; Reich, 2018). Therefore, from a population genomics perspective, even a "thin" sample size of 5 to 10 can be highly informative on the population evolution history.

Reviewer #2: The substantial number of newly sequenced Plasmodium vivax isolates in this dataset is a significant contribution to the malaria research community. However, considering a broader context for analyzing this dataset could potentially enhance the contribution of this work to science. Nevertheless, this study, while incremental, contributes to the field with its data, which are definitively of value.

The rejection of a "naïve" single origin followed by a severe bottleneck model seems compatible with the data. A potential European origin of the populations found in the Americas was previously discussed (Culleton et al. 2011, Taylor et al. 2013). Adding the data from the article by van Dorp that studies a particular isolate using ancient DNA (cited by the authors) was necessary because the original paper was somehow limited in its analyses. However, it is also essential to include the data and work by Michel et al. 2024 (doi.org/10.1038/s41586-024-07546-2). This latest study was excluded from their discussion,

and it reached interesting conclusions as it highlighted more the European postcolonial contacts of a diverse population. Perhaps this work was finished before the Michel et al. 2024 paper became available.

Unfortunately, as presented, the authors seem to reproduce the main results of Michel et al. 2024 with different methods but less data from Europe. The strong geographic population structure exhibited by P. vivax, not only in The Americas but worldwide, is definitively interesting but, again, has been reported by others. The data, however, is of great importance to the malaria research community, so I encourage the authors to address the diversity of P. vivax with a broader scope than just focusing on The Americas.

R2. We are grateful to the reviewer for providing this summary. It should be noted that the article and data by Michel *et al.* (2024) were published in June 2024, whereas our manuscript was submitted in May 2024. We agree with the reviewer that incorporating the data from Michel *et al.* (2024) would be essential for future research, but this is not feasible for us to integrate and reanalyze everything in this paper. Nevertheless, in the revised version of our manuscript, we discuss our results in light of those presented by Michel *et al.* (2024). While the study of Michel *et al.* (2024) brings indeed four new ancient genome samples from Europe (which are of very low coverage and quality overall), their sampling from the rest of the world was way more limited than the one used in our study. For example, their study falls very short in samples from West Africa (they did only have one haploid sample, n=1), and from other places as well. These Western African samples that we included here are critical to understand properly the invasion histories of the Americas by *P. vivax*. As we mentioned in our introduction (and the study of Michel *et al.* (2024) has the same limits), previous studies could not properly assess the potential origins of the South American populations and did make a strong claim about the fact that Europe was the single origin of the *P. vivax* introduction in Latin America. Here we show that even if this is true that Europe significantly contributed, West Africa might also have contributed as well to a more limited level, and our ABC scenario testing (together with other analyses) provides a formal way to test for this. Considering all the above, we do not simply “reproduce” the results that were presented before, since none of them had *P. vivax* samples from West Africa (and very few from other places as well, such as the Middle East).

Reviewer #3: The authors employ complimentary approaches – population genetics (structure, diversity, ancestry) and coalescent based scenario testing to infer the evolutionary history of Plasmodium vivax in Latin America with particular emphasis on when and from where it most likely migrated to the region. A number of other studies using mitochondrial or microsatellite markers have addressed this question, variously implicating Melanesia, Africa, Europe, and South-Asia. The current study employs an expanded number of P. vivax genomes (620), including isolates from West Africa, and one ancient DNA sample from Spain. The authors conclude that the current P. vivax populations in Latin America primarily originated from an extinct European lineage, as well as an unsampled population, which they conclude to be of likely West African origin.

In general the population genetics analyses are thorough and provide a strong basis for the conclusion that Latin American P. vivax forms a distinct group without strong evidence of admixture with other regional populations, and that the Ebro isolate most closely resembles the Latin American population. Divergence time estimates between Latin American and

other populations are most recent for the Ebro sample and West Africa. Twelve ancestry scenarios featuring the Ebro isolate, Mauritanian, and Colombian populations were tested using a simulation-based approximate Bayesian computation (ABC) statistical framework that relies on the Random Forest tree classifier (ABC-RF).

Some results have been over-interpreted (see specific comments below). Additionally, the organizational structure of the manuscript could be improved. Arguments and conclusions are repeated in great detail in several sections (introduction, Results, Discussion) of the manuscript. In particular, the Discussion section could be condensed and several subheadings removed.

R3. We thank the reviewer for his summary. We have taken his/her recommendations into account, ensuring that there was no over-interpretation of our results, and reorganized the article following his/her suggestions. We provide further details on how we addressed the reviewer's comments in the dedicated section below.

Part II – Major Issues: Key Experiments Required for Acceptance

Please use this section to detail the key new experiments or modifications of existing experiments that should be absolutely required to validate study conclusions.

Generally, there should be no more than 3 such required experiments or major modifications for a "Major Revision" recommendation. If more than 3 experiments are necessary to validate the study conclusions, then you are encouraged to recommend "Reject".

Reviewer #1: My central contention with the paper is that given the limitations of sampling, and the weakness of the signals they are attempting to measure, the authors cannot refute the most parsimonious and simple hypothesis that P. vivax in the Americas is derived almost exclusively from the extirpated European population, despite suggestive language to the contrary in the title and throughout. As noted by the authors around line 263, the EBRO sample from Spain dating from the 1940s shows a similarly complex potential ancestry as the Latin American samples by the PCAngsd analysis in Figure 2c. If the authors cannot address the possibility that the European source population (if typified by EBRO) was already admixed, it seems unwarranted to speculate that there was a 'complex journey' of Pv to Latin America. All the parasites could have come from Europe. Period. This possibility is acknowledged again by the authors in the context of the f3 analysis on line 308, but not given adequate attention as a parsimonious scenario in the discussion, which focuses on insights from ABC-RF and other fancy approaches. Further, the admixture signal from W. Africa in Figure 2c appears much stronger at K=6 than K=9. It is unclear to what degree the central novel result of this paper is driven by the fragile choice of a K value.

R4. We thank the reviewer for his comment. We would like first to underline that the signal of admixture from West Africa to Latin America recovered in the Admixture analysis of PCAngsd at K=6, but also at K=9 was not interpreted as definitive evidence of West African contribution to the American gene pool. Rather, it indicated that we could not exclude the

West African population as a potential contributor in the scenarios of the invasion history of the American *P. vivax*. This is why we tested scenarios including West Africa using the simulation-based framework provided by DIYABC-RF.

Regarding the legitimacy of our simulation-based demographic scenario testing using the ABC, many studies (Estoup and Guillemaud, 2010; Lawson et al., 2018; Maier et al., 2023; North et al., 2021) demonstrated that various demographic histories could lead to very similar values or patterns of admixture, *f-statistics* values, and even population graph relationships. We disagree with the reviewer that just looking at admixture patterns or f_3-statistics values is enough to settle on a given scenario. Indeed, these methods are susceptible to be influenced by the presence of multiple sources, some of which may not be sampled. They can also be affected by complex evolutionary events occurring in ancestral populations (structure, migration, change in population size), which are not formally modeled in these approaches. Each method (admixture, *f-statistics* values, and even population graph relationships) captures just a slight fraction of the complex dimensionality of a genomic dataset. The examples discussed in the cited paper above should suffice to legitimate the use of scenario testing to gauge the evidence in support of different scenarios and assess the power of the data to discriminate among different scenarios. The simulation-based ABC scenario testing framework not only uses simulations but also many summary statistics (including the f_3-statistics, diversity, and differentiation metrics) to assess which invasion scenarios most likely resemble to our observed dataset. In our opinion, this ABC approach provides a much more accurate representation of what we suppose are the most probable scenarios among those that are tested.

So, we tested 12 scenarios with DIYABC-RF, some of which included unsampled populations and/or admixture events. We haven't ruled out the possibility of a purely European origin, as this is tested with scenario 2 (Figure 5). And yet, this scenario was not the best one. It accounted for only 6.6% of the votes on average. Other scenarios provided a much better fit to the data: scenarios 4, 6, and 8 (with 14.4%, 18.4%, and 13.2% of votes, respectively). Scenarios 6 and 8, in particular, include an unsampled population (Figure 5), suggesting that the history of *P. vivax* in Latin America is more complex than a single introduction from Europe. Now of course, further investigation is needed to identify the precise origin of this unsampled population, but these results clearly show that an invasion history is not as simple as currently depicted in the literature. We cannot interpret at first glance the summary statistics, without formally quantifying how well they support a scenario better than another one.

Following the reviewer's recommendations, we have modified the title and provided additional clarification regarding the new central result, which is not solely based on the K value (lines 457-463).

The authors make the valid point that the EBRO data are a composite from multiple specimens, and are of poor quality. However, the recent publication of the ancient DNA analysis by Michel and colleagues in Nature last week fortifies the signal from EBRO, and unfortunately renders much of the text in the Introduction and Discussion sections of this manuscript in need of updating. The Michel manuscript finds strong affinity between EBRO, ancient European Pvivax genomes, contemporary P.vivax genomes from the Americas, as

well as a Pvivax genome from Peru that dates back 500 years. The Michel manuscript worked with previously published genomes from Mauritania, Madagascar, and Eritrea (as well as Ethiopia) but found no evidence of admixture in European/American Pv genomes. This disparity in outcomes should be addressed by the authors. Is it a product of fewer W. African genomes in the Michel analysis? Or fewer genomes from French Guiana, Guyana, and Venezuela? Or ?

R5. The article by Michel *et al.* (2024) was published in June 2024, after our study was submitted (May 2024). We value a lot the new insights that were brought by the four new ancient DNA *P. vivax* samples that were brought by that study. Thus, we have rewritten the introduction (lines 123-132 and lines 142-147) and discussion (lines 663-665, 689-695, and 704-716) to take their findings into account. Indeed, Michel *et al.* (2024) manuscript did not find evidence of West African admixture in European and American *P. vivax* genomes, based on their four new samples and the ~5000 SNPs they were able to retrieve. Now this lack of admixture signal could be certainly explained by the fact that their study only included **one** sample from West Africa, which may prevent confirming or refuting the contribution of this region to the American *P. vivax* gene pool. A sample size of n=1 haploid genome does not allow any clustering analysis like ADMIXTURE or PCAngsd to properly model the allele frequency of the West African cluster. Thus any potential West African contribution would be ignored by Michel *et al.* (like previous studies). Even a PCA would be extremely problematic in properly representing the axes of genetic variation with only one sample from a distinct genetic pool like West Africa (as explained by McVean (2009)). Thus this is not surprising that our results differ from those of Michel *et al.* (2024) and also previous studies as well, since only one Mauritanian sample was previously available. This is one of the main reasons why our new sampling provides significant advances to the field since our study brings for the first time enough samples (n=15) to test all contemporaneous potential sources that could have contributed to the genetic makeup of the Latin American populations. Our ABC analyses also allowed accounting for unsampled or extinct populations, which turned out to be important in unraveling the invasion history of *P. vivax* in the Latin Americas.

Now, the overall genetic structure of *P. vivax* is identical between our study and Michel *et al.* (2024), so if redoing all the genetic analyses we performed (which is logistically not possible at this point), we would expect to find the same results even with the addition of these four newly sequenced European samples, with highly degraded DNA for which only ~5000 SNPs could be recovered.

Specific Points:

As noted above, the introduction and discussion (if not the dataset and analysis itself) need significant revision in light of the Michel paper for this paper to maintain relevance. For example, the first sentence of the Conclusion section (that Pv was introduced to the Americas in the 19th century) is now strongly unsupported by the data from the 500 year old Chachapoyas (Peru) sample in the Michel paper. The analyses underlying this conclusion, and their underlying assumptions, should be revisited.

The title and language used to describe the origin of the American Pvivax population should be toned down further, unless the evidence for a complex journey can be strengthened.

R6. We agreed with the reviewer and we revised our manuscript in light of the study of Michel *et al.* (2024) in the introduction (lines 123-132 and lines 142-147) and the discussion (lines 663-665, 689-695, and 704-716).

Line 611: under what plausible historical human scenario could the European, West African, and 'unsampled' P.vivax populations have all diverged at the same time (400 years ago)? Could this imply that W. African Pv populations were also introduced from Europe during the colonial era? The ABC-RF analysis feels over-interpreted given the light depth of sampling of many regions and the departures of plasmodium from Wright-Fisher expectations (including generations that occur without outcrossing when monoclonal infections are transmitted, which should be mentioned)

R7. Thank you for this question. As suggested by the reviewer, it is indeed possible that the transatlantic trade (from the 15th to the 19th centuries) brought the European and West African populations of *P. vivax* into contact, which could explain the trifurcation 400 years ago suggested by our results. The reviewer is right to point out that the estimated time may be biased due to the biological specificity of *P. vivax* which departs from a "simple" Wright-Fisher model used in our ABC simulations. We discuss this in more detail in the Discussion (lines 757-764).

Reviewer #2: Overall, the article offers limited new insights by focusing just on the origin of P. vivax in the Americas. The sampling from the Americas, Africa, and Asia corroborates previous findings of strong population structure. However, the paper does not include data from potential African areas where vivax could have been imported into the Americas nor populations that could have contributed to the now-extinct European populations limiting their capacity to explore complex colonization scenarios. Thus, the statement that this work "represents nearly all potential source populations worldwide" is inaccurate and the limitation of the sampling should be acknowledged.

R8. We acknowledge that the current *P. vivax* sampling does not include all potential African areas that may have contributed to the introduction of *P. vivax* into Latin America: Angola, for example, where one of the main ports of the transatlantic slave trade was located. As highlighted (lines 674-681), this limitation is primarily due to the availability of *P. vivax* samples from Africa at the time of our study. We thus made an effort to include the most comprehensive dataset available, which includes populations from West Africa, where *P. vivax* circulation has been documented.

Regarding the extinct European *P. vivax* populations, we aimed to address this by including populations from the Middle East (Armenia, Azerbaijan, Iran, and Afghanistan), which could potentially have connections to these now-extinct strains. We agree that this limitation should be acknowledged, and we revised the manuscript to reflect better the constraints of our sampling and clarify that while our work includes the most comprehensive dataset currently available, it does not fully encompass all potential source populations (in the abstract and lines 162, 579, 674-681 and 789-793). We thus modified the text accordingly, and moderated our writing by "represents nearly all the potential source populations worldwide, for which genomic data was currently available".

Considering the impact of PloS Pathogens, the data from Michel et al. 2024 must be included in any analysis. I see no convincing support for a West African contribution, so I suggest the authors elaborate more. The conclusion seems to have limited backing from data and more from the assumptions and scenarios tested in the model. For example, they could put the magnifier in other hypothetical colonizations, such as including populations like Ethiopia or India, and see how the models behave. Also, it is important to include others from The Americas, like Brazil and Guyana, not just Colombia (the analysis with DIYABC-RF is interesting but also the methodology from Speidel et al. allows us to address migration, perhaps comparing results is of interest). If they find the colonization from West Africa, then the result will be more robust; my concern is that they are observing connections in the universe of samples they choose to model, which may not capture reality. “Europe” could have been particularly isolated from other areas; but there was commerce, so there could have been gene flow and population structures in pre-colonial times. Most of those contacts with Europe were excluded from the colonization pathway (the focus on the Americas perhaps biased the big picture). Considering that Europe has such importance, the data from Michael et al. 2024 is crucial.

R9. We thank the reviewer for this comment. We repeated the DIYABC-RF analysis for scenario selection using this time the population from French Guiana instead of Colombia. Among the 12 scenarios tested, scenario 6 remained the most likely, receiving an average of 21.90% support (see Table S4). This finding further supports the conclusions of this study regarding the history of *P. vivax* invasion in Latin America, and it was added to the manuscript (lines 515-542). We agree that future studies should include the data of Michel *et al.* (2024), but this is not feasible in the present study. The reviewer should keep in mind that including the data from Michiel *et al.* (2024) would imply redoing all the analyses while adding four ancient highly degraded samples of poor quality for which only 5000 SNPs could be recovered. Our manuscript was submitted before the publication of this data and the first author was at the end of her thesis. She will start a new postdoc with the authors of the team that published that manuscript in Nature. This is their perspective to do what the reviewer is suggesting while expanding the population genetic analyses that will be done (including the Speidel et al approach mentioned by the reviewer elsewhere). While we agree to update our manuscript to account for the new finding brought by Michel *et al.* (2024), this is not reasonable at this time to redo all the analyses in the present manuscript. Furthermore, Michel *et al.* (2024) show that the European samples form a single gene pool, of which Ebro could be a representative. The overall structure of *P. vivax* is also identical between our study and Michel et al. (2024), so we would expect to find the same results even with the addition of these 4 newly sequenced ancient samples from Europe. This is even truer since these are ancient DNA, with very low coverage and highly degraded. This means the number of SNPs they analyzed was small (5k SNPs), and the low-quality SNPs may not be very informative. Now even if we cannot redo all the genomic analysis as explained above, we took into consideration the paper of Michel in the revised version of our manuscript. Regarding all the scenarios testing adding Ethiopia or India, it is clearly out of the scope of the present study.

What is clear, from the strong population structure reported here and elsewhere, is that the

separation of The Americas was expected and comparable with the separation of Southeast Asia from Africa, the Indian subcontinent, etc. The limited data from Bangladesh seems separated from India, but India is a subcontinent, so “India” as a geographic location is misleading. The same applies to Brazil and Colombia, which have complex geographic landscapes and highly structured populations. Some areas of the Amazonia in Brazil could be easily connected with Peru, Colombia, and Ecuador. In contrast, others are more clearly associated with Venezuela and Guyana because of gold mining in the Guyana Shield. The Pacific Coast of Colombia is expected to relate to Central America. Perhaps focusing on other results can enrich the impact of the paper.

R11. We would like to thank the reviewer for this comment. Indeed, each country is a mosaic of different environments, and classifying samples by country may be misleading. Nevertheless, around a third of our samples (n=199), mostly those from Latin America, had no geographical information more precise than the country level, notably because these samples came from travelers and samples were bio-referenced in Reference National Centers as it is. This is for this reason that we had to remain at this level of geographical precision for all samples in this study. Indeed, discussing other results might be relevant, yet it may also dilute the main message and purpose of this study. Furthermore, it is difficult to add any new analyses as the first author has now finished her PhD.

All extant vivax populations appear to be relatively modern with these analyses; maybe the authors could elaborate. The rationale for the mutation rate and number of generations per year need to be clarified since it determines the time estimates, perhaps exploring other scenarios is important. The method developed by Speidel et al. allows for testing scenarios with different mutation rate estimates, etc. However, I'm not sure how the hypnozoite affects the modeling framework implemented in terms of overlapping generations. Perhaps some simulated data could be used to test its impact on the estimates yielded by the methods.

R12. We thank the reviewer for this remark. Indeed, based on our analyses, all current (*extant*) *P. vivax* populations appear to have emerged or evolved relatively recently in evolutionary terms. The relatively recent demographic expansions observed in *P. vivax* populations may result from historical population bottlenecks, possibly linked to climate changes, human population movements, or selective pressures such as immunity development in host populations. This has been shown in previous studies that *P. vivax* populations underwent expansions following these bottleneck events (Arnott et al., 2012; Hupalo et al., 2016). In addition, the global spread of *P. vivax* appears closely tied to human migrations, particularly the colonization of the Americas and the Indian Ocean. Genetic evidence suggests that *P. vivax* was introduced into Latin America relatively recently via colonization. This aligns with the relatively shallow divergence times observed in extant *P. vivax* populations from Africa, the Americas, and Asia, and this is also supported by studies like van Dorp *et al.* (2020). As suggested by the reviewer we added some sentences about this point (lines 393-397). Mutation rate and generation time were chosen from the literature (Daron *et al.* 2021, based on *Plasmodium falciparum* cultured in vitro in human erythrocytes in Claessens *et al.* (2014)). Although it is used in the coalescent approach of *Relate* and *Colate*, no mutation rate is set by the user directly for DIYABC-RF, since it simulates SNP data based on the approach Hudson (2002) (cf –s 1 option in the program *ms* associated to Hudson (2002)). Nevertheless, these analyses delivered consistent divergence times. Even

if it is far from perfect, these results suggest that this mutation rate may be an adequate approximation for *P. vivax*, which is underlined in lines 735-746.

Furthermore, as the reviewer points out, dormancy in *P. vivax* can affect inferences. Indeed, it would overestimate population sizes and underestimate divergence times. This is discussed in lines 747-756.

On a side note, our manuscript used the approaches of Spiedel (https://leospeidel.github.io/research/) which are *Relate* and *Colate* (for ancient DNA). This approach allowed us to estimate coalescent rates within each group, and also estimated change in *Ne* back to the TMRCA. At the moment, they do not offer scenario testing procedures similar to the one used in the ABC approach, and this is not the scope of the present study to develop a new one.

Reviewer #3: (No Response)

Part III – Minor Issues: Editorial and Data Presentation Modifications
Please use this section for editorial suggestions as well as relatively minor modifications of existing data that would enhance clarity.

Reviewer #1: Small points
Extra use of the word 'countries' in line 126
Corrected on line 126.

Reviewer #2: The data is important but also is being self critical of its limitations. Data from the middle East is lacking still so encouraging the readers toward exploring the sampling gaps could be of interest. It is also an overstatement to say that all potential sources are considered since the Gordian knot seems to be Europe.
We thank the reviewer for this comment. We have added a paragraph to encourage future studies to fill the sampling gap in the Middle East and Europe (lines 789-793). We have also toned down the statements about considering all sources (in the abstract and lines 162 and 579), as explained in our answer R8.

Please provide the bioproject number in the maintext.
The bioproject and the accession numbers have been added in the main text (line 1112) and the S1 supplementary table.

Reviewer #3: Line 65. The authors state that "knowledge of when and from where P. vivax colonized Latin America is "necessary to develop efficient strategies for malaria control and elimination and for monitoring its spread in new regions" yet the rationale for this statement is lacking and it seems to be a bit of a stretch.
We would like to thank the reviewer for this insightful comment. Understanding the origins of *P. vivax* in Latin America is indeed crucial for several reasons. First, this knowledge is essential for studying how *P. vivax* adapts to new environments and this can inform predictions about its adaptation in Latin America or other regions and guide tailored control

strategies. This knowledge is precious for tailoring region-specific control strategies. Additionally, insights into its historical movement can help anticipate its potential spread to new areas due to factors such as migration or climate change. This might be helpful in the preparation for outbreaks in regions where malaria is not yet prevalent. We added in the manuscript some sentences to illustrate better the rationale for this statement (lines 87-91)

Line160-169. More appropriate in the Methods section.
We thank the reviewer for this comment. This paragraph was moved to the Materials and Methods section (lines 949-965).

Figure 2a.
•A 3-axis PCA plot without animation (rotation) is difficult to interpret visually.
We have chosen to show the 3D PCA in Figure 2a in order to visualize the four genetic groups that are also described in the maximum likelihood tree and ancestry plots. To visualize the 2D PCA, please refer to the S4 figure.

•Labels – Cameroon and Central Africa are grouped with Mauritania under the heading West Africa and yet they are central African.
We thank the reviewer for his comment. This has been modified in Figure 2a-b.

•Not clear why only 105,527 unlinked SNPs used rather than 950,034
We thank the reviewer for this comment. This is explained in detail in the Materials and Methods section: "The PCA and the ancestry plots were computed with ANGSD and PCAngsd v0.98, after selecting only biallelic SNPs present in the core region of the *P. vivax* genomes and excluding SNPs with a minor allele frequency (MAF) ≤5%. The variants were LD-pruned to obtain a set of unlinked variants using ngsld v1.1.1, with a threshold of r^2=0.5 with windows of 0.5kb. In total, 105,527 SNPs were used for 620 individuals (*P. vivax-like* samples were removed)" (lines 980-985). The application of LD-pruning is important to include only physically unlinked SNPs in the PCA, so that the variance depicted is only impacted by population structure and demographic history. Indeed, low recombining regions in the genome may significantly bias the PCA (for example, selection and inversion). However, as the reviewer noticed a lack of clarity, we have added information to lines 226-227.

Figure 2d. If K=6 is the best K-value based on the broken-stick eigenvalues plus one method, why does 2d illustrate K=9?
As this study focuses on Latin America, figure 2D shows K=9 to show the reader the substructure in Latin America, which is only visible from K=9 (as described in the text line 253). However, figure 2c shows K=6, the optimum K as well. Note also that estimating the optimum number of K is also to be taken with caution (Janes et al., 2017; Pritchard et al., 2000), and that it is advisable to study several values of K (Lawson et al., 2018).

Line 245. Results from PC4 are discussed but this PC only explains 2.28% of the variation.

Although PC4 explains only 2.28% of the variation, it remains relevant for analysis according to the broken-stick eigenvalues method (Frontier, 1976). This suggests that the first 5 PCs are relevant to study to understand the structure of global *P. vivax* populations. Furthermore,

this PC suggested a pattern that had already been reported before in the Malaysian region.

Line 303-311. It is not clear what the admixture fs-statistics add to the analysis as the results are inconclusive.

The f_3-statistics suggest no recent significant admixture involving American populations, which aligns with the population networks inferred by TreeMix and AdmixtureBayes. While these results are not conclusive, they are consistent with other analyses. This allowed us to conclude that there was no trace of recent admixture in the Latin American populations.

Line 394. "These divergence time estimates are consistent with the population branching order from the previous analyses (Fig. 2b and 3)". Figure 2b does not provide strong basal branch support and therefore cannot be said to be consistent with divergence time estimates.
The remark is pertinent and we have removed this sentence from the text (line 444).

The authors acknowledge that the population genetics analyses provide only weak support for a West African contribution to genetic ancestry of P. vivax populations in Latin America (Fig 2C).

We would like to thank the reviewer for this comment but we are not sure we understand the expected answer to it.

Colonization history scenarios. Why was the Colombian population chosen? Do the results differ if different Latin American populations are used.
We thank the reviewer for this question and comment. The Colombian population was chosen because it represented the non-admixed population from Latin America with the largest sample size (n=30). We have clarified and justified this choice in the manuscript (lines 485-487). On the other hand, the population with the most individuals in the other American subgroup is French Guiana (n=14). As explained in answer R9, we repeated the DIYABC-RF analysis considering French Guiana instead of Columbia and the best-fitting scenario remained the same (scenario 6). We described the results above in R9 and in the manuscript (lines 515-542, also see S4 Table).

Line 453. What were the mean posterior probabilities of scenarios 4 and 8?
We thank the reviewer for this question. As explained by Pudlo *et al.* (2016): in DIYABC-RF, the model choice is done as a classification problem using a supervised machine learning tool named Random Forest (RF) that works in 2 steps: (1) predicting the model that best fits the data with RF classification votes for each scenario tested (there is thus a number of votes for each scenario tested), and then (2) the approximation of the posterior probability is conducted only on the selected model during a second step also relying on RF. The posterior probability and its error rate are thus only computed for the scenario that receives the highest number of votes (scenario 6 in this case). Please see Pudlo *et al.* (2016) and also Sisson *et al.* (2018) for further details.

Fig 3. Other than highlighting the distinctness of the Latin American populations, what does this analysis add?

We would like to thank the reviewer for this question. As the reviewer correctly notes, this analysis clearly shows that American populations have not recently mixed with populations from the rest of the world. This analysis also suggests that there are no interbreeding events between American populations. While this is not the primary focus of our study, it provides valuable insights into the relationships between *P. vivax* populations globally. For example, this has enabled us to highlight an admixture event between Ebro and Oceanian populations, which is discussed in the manuscript (lines 640-652).

Fig 5. Labels are not defined. What is Nbot?

We thank the reviewer for this comment. Labels starting with N correspond to effective population sizes (solid lines in Figure 5). The names of the labels are arbitrary, and the distributions of each parameter are detailed in the S3 Table. We have clarified this in the legend of Figure 5.

Line 467. Given the large 90% confidence interval associated with estimated divergence it seems reporting specific years for the divergence times is a bit of an over-interpretation.

We thank the reviewer for this comment. As these are only average estimated divergence times, we add this specification in the paragraph (lines 546-557).

Discussion

This section would benefit from significant editing. As it is, many of the arguments and results reported in previous sections (Introduction, Results) are repeated here. A clear synthesis of the salient results would be preferable.

We thank the reviewer for this comment. We have rewritten the discussion to shorten it and avoid repetition.

References cited in the answers to reviewers:

Arnott, A., Barry, A.E., Reeder, J.C., 2012. Understanding the population genetics of Plasmodium vivax is essential for malaria control and elimination. Malar. J. 11, 14. https://doi.org/10.1186/1475-2875-11-14

Claessens, A., Hamilton, W.L., Kekre, M., Otto, T.D., Faizullabhoy, A., Rayner, J.C., Kwiatkowski, D., 2014. Generation of Antigenic Diversity in Plasmodium falciparum by Structured Rearrangement of Var Genes During Mitosis. PLOS Genet. 10, e1004812. https://doi.org/10.1371/journal.pgen.1004812

Daron, J., Boissiere, A., Boundenga, L., Ngoubangoye, B., Houze, S., Arnathau, C., Sidobre, C., Trape, J.-F., Durand, P., Renaud, F., Fontaine, M.C., Prugnolle, F., Rougeron, V., 2021. Population genomic evidence of Plasmodium vivax Southeast Asian origin. Sci. Adv. 7, eabc3713. https://doi.org/10.1126/sciadv.abc3713

Estoup, A., Guillemaud, T., 2010. Reconstructing routes of invasion using genetic data: why, how and so what? Mol. Ecol. 19, 4113–4130. https://doi.org/10.1111/j.1365-294X.2010.04773.x

Frontier, S., 1976. Étude de la décroissance des valeurs propres dans une analyse en composantes principales: Comparaison avec le moddle du bâton brisé. J. Exp. Mar. Biol. Ecol. 25, 67–75. https://doi.org/10.1016/0022-0981(76)90076-9

Hahn, M.W., 2018. Molecular Population Genetics. Oxford University Press.

Hudson, R.R., 2002. Generating samples under a Wright–Fisher neutral model of genetic variation. Bioinformatics 18, 337–338. https://doi.org/10.1093/bioinformatics/18.2.337

Hupalo, D.N., Luo, Z., Melnikov, A., Sutton, P.L., Rogov, P., Escalante, A., Vallejo, A.F., Herrera, S., Arévalo-Herrera, M., Fan, Q., Wang, Y., Cui, L., Lucas, C.M., Durand, S., Sanchez, J.F., Baldeviano, G.C., Lescano, A.G., Laman, M., Barnadas, C., Barry, A., Mueller, I., Kazura, J.W., Eapen, A., Kanagaraj, D., Valecha, N., Ferreira, M.U., Roobsoong, W., Nguitragool, W., Sattabonkot, J., Gamboa, D., Kosek, M., Vinetz, J.M., González-Cerón, L., Birren, B.W., Neafsey, D.E., Carlton, J.M., 2016. Population genomics studies identify signatures of global dispersal and drug resistance in Plasmodium vivax. Nat. Genet. 48, 953–958. https://doi.org/10.1038/ng.3588

Janes, J.K., Miller, J.M., Dupuis, J.R., Malenfant, R.M., Gorrell, J.C., Cullingham, C.I., Andrew, R.L., 2017. The K = 2 conundrum. Mol. Ecol. 26, 3594–3602. https://doi.org/10.1111/mec.14187

Lawson, D.J., van Dorp, L., Falush, D., 2018. A tutorial on how not to over-interpret STRUCTURE and ADMIXTURE bar plots. Nat. Commun. 9, 3258. https://doi.org/10.1038/s41467-018-05257-7

Maier, R., Flegontov, P., Flegontova, O., Isildak, U., Changmai, P., Reich, D., 2023. On the limits of fitting complex models of population history to f-statistics. eLife 12, e85492. https://doi.org/10.7554/eLife.85492

McVean, G., 2009. A Genealogical Interpretation of Principal Components Analysis. PLOS Genet. 5, e1000686. https://doi.org/10.1371/journal.pgen.1000686

Michel, M., Skourtanioti, E., Pierini, F., Guevara, E.K., Mötsch, A., Kocher, A., Barquera, R., Bianco, R.A., Carlhoff, S., Coppola Bove, L., Freilich, S., Giffin, K., Hermes, T., Hiß, A., Knolle, F., Nelson, E.A., Neumann, G.U., Papac, L., Penske, S., Rohrlach, A.B., Salem, N., Semerau, L., Villalba-Mouco, V., Abadie, I., Aldenderfer, M., Beckett, J.F., Brown, M., Campus, F.G.R., Chenghwa, T., Cruz Berrocal, M., Damašek, L., Duffett Carlson, K.S., Durand, R., Ernée, M., Fântăneanu, C., Frenzel, H., García Atiénzar, G., Guillén, S., Hsieh, E., Karwowski, M., Kelvin, D., Kelvin, N., Khokhlov, A., Kinaston, R.L., Korolev, A., Krettek, K.-L., Küßner, M., Lai, L., Look, C., Majander, K., Mandl, K., Mazzarello, V., McCormick, M., de Miguel Ibáñez, P., Murphy, R., Németh, R.E., Nordqvist, K., Novotny, F., Obenaus, M., Olmo-Enciso, L., Onkamo, P., Orschiedt, J., Patrushev, V., Peltola, S., Romero, A., Rubino, S., Sajantila, A., Salazar-García, D.C., Serrano, E., Shaydullaev, S., Sias, E., Šlaus, M., Stančo, L., Swanston, T., Teschler-Nicola, M., Valentin, F., Van de Vijver, K., Varney, T.L., Vigil-Escalera Guirado, A., Waters, C.K., Weiss-Krejci, E., Winter, E., Lamnidis, T.C., Prüfer, K., Nägele, K., Spyrou, M., Schiffels, S., Stockhammer, P.W., Haak, W., Posth, C., Warinner, C., Bos, K.I., Herbig, A., Krause, J., 2024. Ancient Plasmodium genomes shed light on the history of human malaria. Nature. https://doi.org/10.1038/s41586-024-07546-2

North, H.L., McGaughran, A., Jiggins, C.D., 2021. Insights into invasive species from whole-genome resequencing. Mol. Ecol. 30, 6289–6308. https://doi.org/10.1111/mec.15999

Pritchard, J.K., Stephens, M., Donnelly, P., 2000. Inference of Population Structure Using Multilocus Genotype Data. Genetics 155, 945–959. https://doi.org/10.1093/genetics/155.2.945

Pudlo, P., Marin, J.-M., Estoup, A., Cornuet, J.-M., Gautier, M., Robert, C.P., 2016. Reliable ABC model choice via random forests. Bioinformatics 32, 859–866. https://doi.org/10.1093/bioinformatics/btv684

Reich, D., 2018. Who We are and how We Got Here: Ancient DNA and the New Science of the Human Past. Oxford University Press.

Sisson, S.A., Fan, Y., Beaumont, M., 2018. Handbook of Approximate Bayesian Computation. CRC Press.

van Dorp, L., Gelabert, P., Rieux, A., de Manuel, M., de-Dios, T., Gopalakrishnan, S., Carøe, C., Sandoval-Velasco, M., Fregel, R., Olalde, I., Escosa, R., Aranda, C., Huijben, S., Mueller, I., Marquès-Bonet, T., Balloux, F., Gilbert, M.T.P., Lalueza-Fox, C., 2020. Plasmodium vivax Malaria Viewed through the Lens of an Eradicated European

Strain. Mol. Biol. Evol. 37, 773–785. https://doi.org/10.1093/molbev/msz264
